# Supplementary material for: COVID-19 patients share common, corticosteroid-independent features of impaired host immunity to pathogenic molds
Source: Front Immunol. 2022 Aug 16;13:954985. doi: 10.3389/fimmu.2022.954985 (PMC9427195; doi:10.3389/fimmu.2022.954985)
Supplement: Supplementary file 1 [file DataSheet_1.pdf]

COVID-19 patients share common, corticosteroid-independent features of impaired host immunity to pathogenic molds

Supplementary Methods

Beeke Tappe[1,#] and Chris D. Lauruschkat[1,#], Lea Strobel[1], Jezreel Pantaleón García[2], Oliver Kurzai[3,4], Silke Rebhan[1], Sabrina Kraus[1], Elena Pfeuffer-Jovic[5], Lydia Bussemer[1], Lotte Possler[6], Matthias Held[5], Kerstin Hünniger[3,4], Olaf Kniemeyer[4], Sascha Schäuble[4], Axel A. Brakhage[4,7], Gianni Panagiotou[4], P. Lewis White[8], Hermann Einsele[1], Jürgen Löffler[1, $,*] and Sebastian Wurster[9,*]

[1]Department of Internal Medicine II, University Hospital of Würzburg, Würzburg, Germany

[2]Department of Pulmonary Medicine, The University of Texas MD Anderson Cancer Center, Houston, Texas, USA

[3]Institute for Hygiene and Microbiology, University of Würzburg, Würzburg, Germany

[4]Leibniz Institute for Natural Product Research and Infection Biology–Hans Knöll Institute, Jena, Germany

[5]Missionsärztliche Klinik Würzburg, Würzburg, Germany

[6]Main-Klinik Ochsenfurt, Würzburg, Germany

[7]Department of Microbiology and Molecular Biology, Institute of Microbiology, Friedrich Schiller University, Jena, Germany

[8]Public Health Wales, Microbiology Cardiff, Wales, UK

[9]Department of Infectious Diseases, Infection Control and Employee Health, The University of Texas MD Anderson Cancer Center, Houston, Texas, USA.

[#]These authors share first authorship

[$]Requests for materials should be addressed to Jürgen Löffler (Loeffler_J@ukw.de)

[*]These authors have contributed equally to this work and are shared last and co-corresponding authors.

*** Correspondence:**
Prof. Dr. Jürgen Löffler, Loeffler_J@ukw.de

Asst. Prof. Dr. Sebastian Wurster, stwurster@mdanderson.org

Supplementary Table 1. Preparation of whole blood stimulation tubes for ex-vivo immunoassays.

	Assay	α-CD28	α-CD49d	AfuLy	AfuG	RarLy	RarG	PrS	PHA	RPMI
**Concentration in ready-to-use stimulation tubes**	n/a	2 µg/mL	2 µg/mL	100 µg/mL	1×10^6 AfuG/mL	100 µg/mL	1×10^6 RarG/mL	1.2 nmol	10 µg/mL	*Ad* 500 µL
**Final concentration after injection of 500 µL WB**	n/a	1 µg/mL	1 µg/mL	50 µg/mL	5×10^5 AfuG/mL	50 µg/mL	5×10^5 RarG/mL	0.6 nmol	5 µg/mL	n/a
**Unstimulated control**	AI, Cyt	X	X							X
	II									X
**AfuLy stimulation**	AI, Cyt	X	X	X						X
**AfuG stimulation**	II				X					X
**RarLy stimulation**	AI, Cyt	X	X			X				X
**RarG stimulation**	II						X			X
**PrS stimulation**	AI, Cyt	X	X					X		X
	II							X		X
**AfuLy + PrS stimulation**	AI, Cyt	X	X	X				X		X
**AfuG + PrS stimulation**	II				X			X		X
**Positive control**	AI, II, Cyt								X	X

X indicates that the compound was used for the respective condition.

Abbreviations: AfuLy = *A. fumigatus* mycelial lysate, RarLy = *R. arrhizus* mycelial lysate, CD = cluster of differentiation, n/a = not applicable, PrS = SARS-CoV-2 Protein S (Alpha variant), PHA = phytohemagglutinin, RPMI = Roswell Park Memorial Institute medium, WB = whole blood, AI = adaptive immunity, II = innate immunity, Cyt = multiplex cytokine assay.

Supplementary Table 2. Antibodies used for flow cytometric analyses.

Name	Fluorochrome	Clone	Provider	Catalogue number	Panel(s)
**CD1c (BDCA-1)**	PerCP-Vio700	REA694	Miltenyi Biotec	130-110-538	DC
**CD3**	PE-Vio615	REA613	Miltenyi Biotec	130-114-520	ID
**CD4**	VioGreen	REA623	Miltenyi Biotec	130-113-230	T1, T2
**CD8**	PerCP-Vio700	REA734	Miltenyi Biotec	130-110-682	T1, T2
**CD11b**	APC	REA713	Miltenyi Biotec	130-110-554	GR
**CD14**	APC-Vio770	TÜK4	Miltenyi Biotec	130-113-144	ID, DC
**CD16**	VioGreen	REA423	Miltenyi Biotec	130-113-397	ID, GR
**CD19**	PE	REA675	Miltenyi Biotec	130-113-646	ID
**CD45RA**	PE-Vio615	REA1047	Miltenyi Biotec	130-117-745	T2
**CD56**	FITC	REA196	Miltenyi Biotec	130-114-549	ID
**CD62L**	PE	REA615	Miltenyi Biotec	130-113-625	GR
**CD66b**	PerCP-Vio700	REA306	Miltenyi Biotec	130-119-768	ID, GR
**CD69**	APC-Vio770	REA824	Miltenyi Biotec	130-112-616	T1, T2
**CD83**	FITC/VioBright515	REA714	Miltenyi Biotec	130-110-507	DC
**CD107a (LAMp-1)**	APC/R667	REA792	Miltenyi Biotec	130-111-626	T2
**CD154**	FITC/VioBright515	REA238	Miltenyi Biotec	130-122-800	T1, T2
**CD183 (CXCR3)**	APC	REA232	Miltenyi Biotec	130-120-450	T1
**CD194 (CCR4)**	PE	REA279	Miltenyi Biotec	130-120-456	T1
**CD196 (CCR6)**	PE-Vio615	REA190	Miltenyi Biotec	130-120-459	T1
**CD197 (CCR7)**	PE	REA546	Miltenyi Biotec	130-119-583	T2
**CD253 (TRAIL)**	PE-Vio615	REA1113	Miltenyi Biotec	130-119-286	GR, DC
**CD279 (PD-1)**	PE-Vio770	REA1165	Miltenyi Biotec	130-120-385	T1, T2
**CD284 (TLR4)**	APC	HTA125	Miltenyi Biotec	130-096-236	DC
**Dectin-1**	PE	REA515	Miltenyi Biotec	130-121-993	DC
**DHR 123**	FITC		Sigma-Aldrich	D1054	GR
**HLA-DR**	VioGreen	REA805	Miltenyi Biotec	130-111-948	DC
**Ki-67**	AlexaFluor700	Ki-67	BioLegend	350530	T1, T2
**Viobility 405/452**	PB450/VioBlue		Miltenyi Biotec	130-110-205	ID, T1,T2, GR, DC

Abbreviations: ID = immune cell distribution panel, GR = granulocyte panel, DC = dendritic cells panel, T1 = T cell panel 1, T2 = T cell panel 2. All antibodies/dyes were used at 2% v/v, except for Ki-67 (5% v/v), DHR 123 (1% v/v), and Viobility 405/452 (1% v/v).

Supplementary Table 3. Utilization of blood samples for downstream readouts.

Assay	Flow cytometry					Cytokine assay	
**Panel**	**ID**	**T1 & T2**	**GR & DC**	**T1 & T2**	**GR & DC**	**Multiplex**	**Multiplex**
**Stimulus**	**n/a**	**Afu**	**Afu**	**Rar**	**Rar**	**Afu**	**Rar**
**Figures**	**1C**	**1D-E<br>2C-H**	**4A-C***	**S6**	**5E-F**	**3A-D<br>S1<br>S2**	**5A-D**
**Patient 1 (Alpha)**	X	X	X			X	
**Patient 2 (Alpha)**	X	X	X			X	
**Patient 3 (Alpha)**	X	X	X			X	
**Patient 4 (Alpha)**	X	X	X			X	
**Patient 5 (Delta)**	X	X	X	X		X	
**Patient 6 (Delta)**	X	X	X	X	X	X	X
**Patient 7 (Delta)**	X	X	X	X	X	X	X
**Patient 8 (Delta)**	X	X	X	X	X	X	X
**Patient 9 (Delta)**	X	X	X	X	X	X	X
**Patient 10 (Delta)**	X	X	X	X	X	X	X
**Patient 11 (Delta)**	X	X	X	X	X	X	X
**Patient 12 (Delta)**	X	X	X	X	X	X	X
**Total number of patient samples**	12	12	12	8	7	12	7
**Control 1**	X	X	X *	X	X	X	X
**Control 2**	X	X	X *	X	X	X	X
**Control 3**	X		X *			X	
**Control 4**	X		X *			X	
**Control 5**	X	X	X *	X	X	X	X
**Control 6**	X	X	X *	X	X	X	X
**Control 7**	X	X	X *			X	
**Control 8**	X	X	X *	X	X	X	X
**Control 9**	X	X	X	X	X		X
**Total number of control samples**	9	7	9<br>(8) *	6	6	8	6

Footnotes and abbreviations on following page.

Footnotes and abbreviations for **Supplementary Table 3**:

X indicates that the subject/sample was included in the respective analyses and figures.

* indicates that the sample was additionally used for co-stimulation experiments with *A. fumigatus* lysate and SARS-CoV-2 protein S.

The fungal killing assays (**Fig. 4D** and **Fig. 5G**), performed after the main analysis, utilized all Delta patient samples and an independent cohort of 6 additional control subjects.

Abbreviations: ID = immune cell distribution panel, T1 = T cell panel 1, T2 = T cell panel 2, GR = granulocyte panel, DC = dendritic cells panel, n/a = not applicable, Afu = *Aspergillus fumigatus*, Rar = *Rhizopus arrhizus*.

Gating strategy

Gating strategy to study global and mold antigen-reactive T-cell responses.

Single cells were gated based on SSC-A, FSC-A, and FSC-H properties. Lymphocytes were identified by light scatter properties and dead lymphocytes were excluded by Live/Dead staining. $CD4^+$, $CD8^{bright}$, and $CD8^{dim}$ cells were differentiated.

In panel T1 (**1st figure**), positive cells for activation markers CD154 and CD69, exhaustion marker PD-1, and proliferation marker Ki-67 were gated within each T-cell subpopulation. $CD4^+$ T-helper (Th) cells were then subdivided into Th1 ($CXCR3^+$), Th2 ($CXCR3^-$ $CCR6^-$ $CCR4^+$), and Th17 ($CXCR3^-$ $CCR6^+$ $CCR4^+$) cells. Th-cell polarization was also determined for $CD154^+$, $CD69^+$, $PD\text{-}1^+$ and $Ki\text{-}67^+$ Th cells. Mean fluorescence intensity was determined for all markers.

In panel T2 (**2nd figure**), positive cells for activation markers CD154, CD69, and CD107a, exhaustion marker PD-1, and proliferation marker Ki-67 were gated within each T-cell subpopulation. Effector memory T cells (T_{EM}, $CD45RA^-$ $CCR7^-$), central memory T cells (T_{CM}, $CD45RA^-$ $CCR7^+$), effector T cells (T_{EMRA}, $CD45RA^+$ $CCR7^-$) and naïve T cells (T_N, $CD45RA^+$ $CCR7^+$) were differentiated. Mean fluorescence intensity was determined for all markers.

Gating strategy to determine baseline leukocyte distributions and to study mold antigen-induced activation of dendritic cells and granulocytes (3rd figure).

Debris was excluded by light scatter properties and dead cells were excluded by Live/Dead staining. For determination of leukocyte distributions (performed on unstimulated samples), T cells ($CD19^-$ $CD3^+$) and B cells ($CD19^+$ $CD3^-$) were gated. $CD19^-$ $CD3^-$ cells were further subdivided into monocytes ($CD14^+$) and granulocytes ($CD66b^+$). Natural killer (NK) cells were identified as $CD3^-$ $CD56^+$ and subdivided into $CD56^{dim}$ $CD16^+$ NK cells and $CD56^{bright}$ $CD16^{low}$ NK cells. Granulocytes were identified by CD66 expression and analyzed for ROS, CD253, CD16, CD11b, and CD62L expression. Dendritic cells, identified as $CD1c^+$ cells, were analyzed for Dectin-1, CD253, HLA-DR, CD83, CD284 and CD14 expression. Mean fluorescence intensity was determined for all markers.

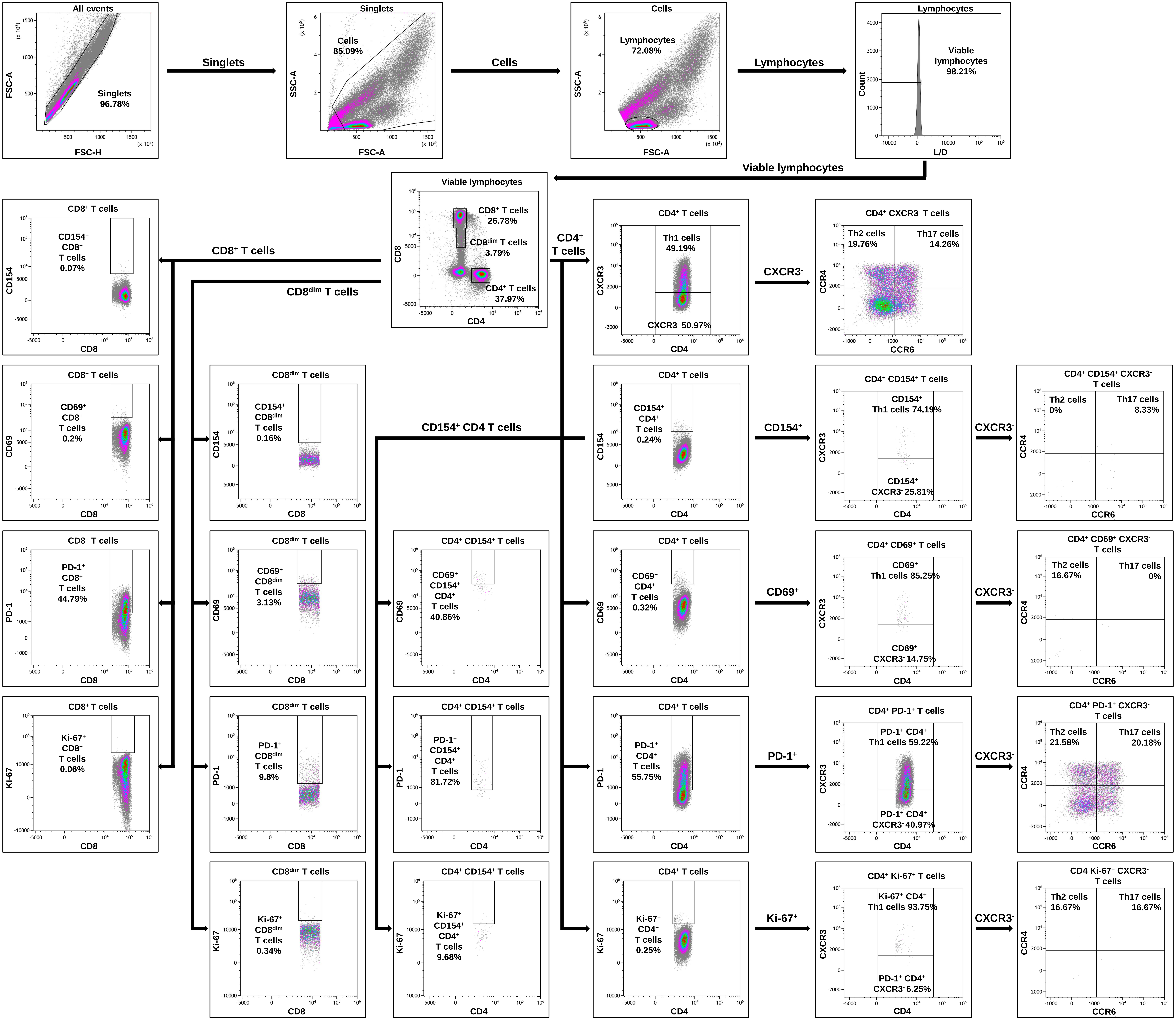
All events
Singlets 96.78%
FSC-A
FSC-H
Singlets
Cells 85.09%
SSC-A
FSC-A
Cells
Lymphocytes 72.08%
Lymphocytes
Viable lymphocytes 98.21%
Count
L/D
Viable lymphocytes
CD8+ T cells 26.78%
CD8dim T cells 3.79%
CD4+ T cells 37.97%
CD8
CD4
CD8+ T cells
CD8dim T cells
CD4+ T cells
CD154+ CD8+ T cells 0.07%
CD154
CD4+ T cells
Th1 cells 49.19%
CXCR3- 50.97%
CXCR3
CXCR3-
CD4+ CXCR3- T cells
Th2 cells 19.76%
Th17 cells 14.26%
CCR4
CCR6
CD69+ CD8+ T cells 0.2%
CD69
CD154+ CD8dim T cells 0.16%
CD154+ CD4 T cells
CD154+ CD4+ T cells 0.24%
CD154+
CD4+ CD154+ T cells
CD154+ Th1 cells 74.19%
CD154+ CXCR3- 25.81%
CD4+ CD154+ CXCR3- T cells
Th2 cells 0%
Th17 cells 8.33%
PD-1+ CD8+ T cells 44.79%
PD-1
CD69+ CD8dim T cells 3.13%
CD69+ CD154+ CD4+ T cells 40.86%
CD69+ CD4+ T cells 0.32%
CD69+
CD4+ CD69+ T cells
CD69+ Th1 cells 85.25%
CD69+ CXCR3- 14.75%
CD4+ CD69+ CXCR3- T cells
Th2 cells 16.67%
Th17 cells 0%
Ki-67+ CD8+ T cells 0.06%
Ki-67
PD-1+ CD8dim T cells 9.8%
PD-1+ CD154+ CD4+ T cells 81.72%
PD-1+ CD4+ T cells 55.75%
PD-1+
CD4+ PD-1+ T cells
PD-1+ CD4+ Th1 cells 59.22%
PD-1+ CD4+ CXCR3- 40.97%
CD4+ PD-1+ CXCR3- T cells
Th2 cells 21.58%
Th17 cells 20.18%
Ki-67+ CD8dim T cells 0.34%
Ki-67+ CD154+ CD4+ T cells 9.68%
Ki-67+ CD4+ T cells 0.25%
Ki-67+
CD4+ Ki-67+ T cells
Ki-67+ CD4+ Th1 cells 93.75%
PD-1+ CD4+ CXCR3- 6.25%
CD4 Ki-67+ CXCR3- T cells
Th2 cells 16.67%
Th17 cells 16.67%

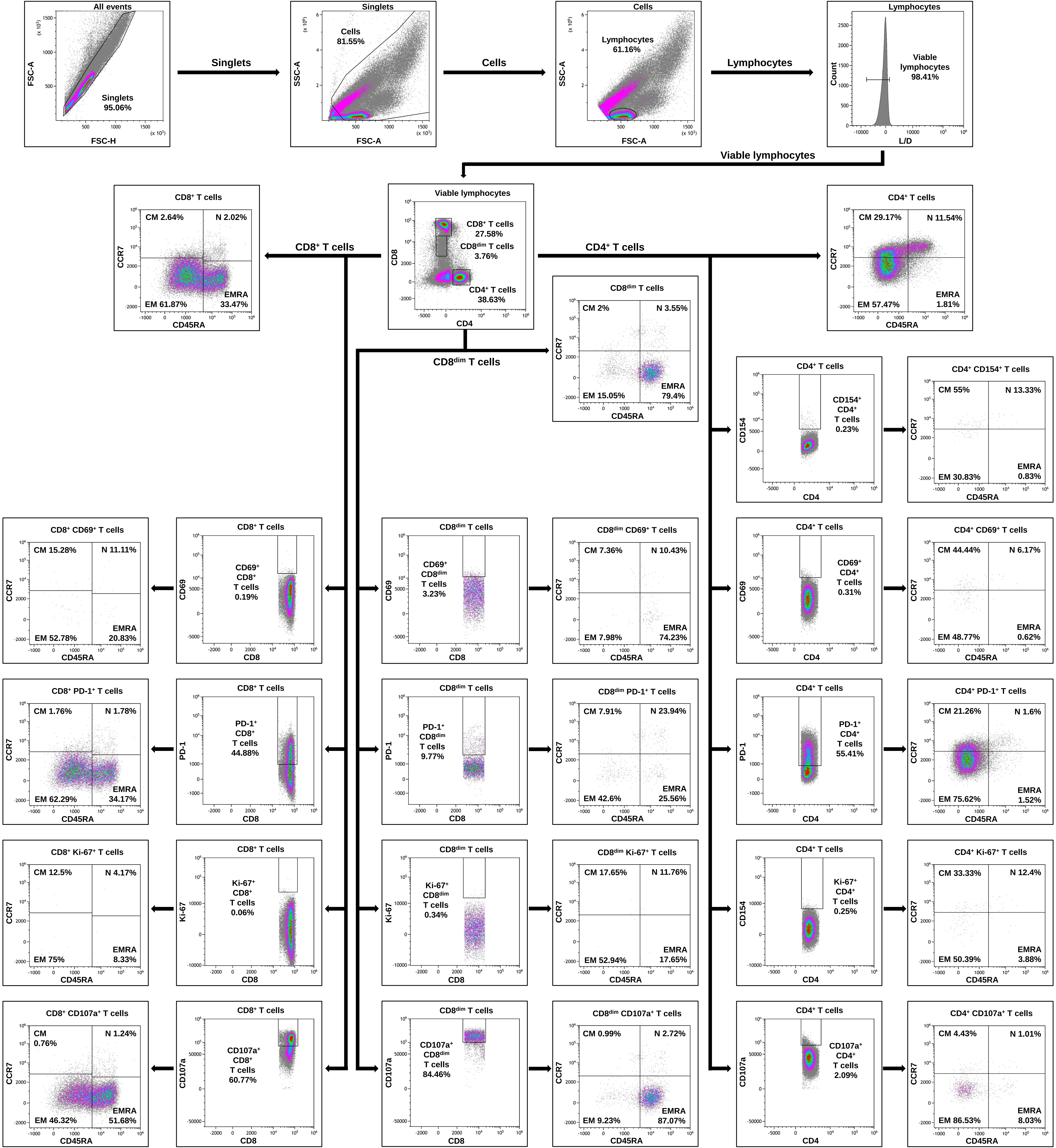
All events
FSC-A
FSC-H
Singlets 95.06%
Singlets
SSC-A
FSC-A
Cells 81.55%
Cells
Lymphocytes 61.16%
Lymphocytes
Count
L/D
Viable lymphocytes 98.41%
Viable lymphocytes
CD8+ T cells 27.58%
CD8dim T cells 3.76%
CD4+ T cells 38.63%
CD8
CD4
CD8+ T cells
CD4+ T cells
CD8dim T cells
CCR7
CD45RA
CM 2.64%
N 2.02%
EM 61.87%
EMRA 33.47%
CM 29.17%
N 11.54%
EM 57.47%
EMRA 1.81%
CM 2%
N 3.55%
EM 15.05%
EMRA 79.4%
CD154
CD154+ CD4+ T cells 0.23%
CD4+ CD154+ T cells
CM 55%
N 13.33%
EM 30.83%
EMRA 0.83%
CD8+ CD69+ T cells
CM 15.28%
N 11.11%
EM 52.78%
EMRA 20.83%
CD69
CD69+ CD8+ T cells 0.19%
CD69+ CD8dim T cells 3.23%
CD8dim CD69+ T cells
CM 7.36%
N 10.43%
EM 7.98%
EMRA 74.23%
CD69+ CD4+ T cells 0.31%
CD4+ CD69+ T cells
CM 44.44%
N 6.17%
EM 48.77%
EMRA 0.62%
CD8+ PD-1+ T cells
CM 1.76%
N 1.78%
EM 62.29%
EMRA 34.17%
PD-1
PD-1+ CD8+ T cells 44.88%
PD-1+ CD8dim T cells 9.77%
CD8dim PD-1+ T cells
CM 7.91%
N 23.94%
EM 42.6%
EMRA 25.56%
PD-1+ CD4+ T cells 55.41%
CD4+ PD-1+ T cells
CM 21.26%
N 1.6%
EM 75.62%
EMRA 1.52%
CD8+ Ki-67+ T cells
CM 12.5%
N 4.17%
EM 75%
EMRA 8.33%
Ki-67
Ki-67+ CD8+ T cells 0.06%
Ki-67+ CD8dim T cells 0.34%
CD8dim Ki-67+ T cells
CM 17.65%
N 11.76%
EM 52.94%
EMRA 17.65%
Ki-67+ CD4+ T cells 0.25%
CD4+ Ki-67+ T cells
CM 33.33%
N 12.4%
EM 50.39%
EMRA 3.88%
CD8+ CD107a+ T cells
CM 0.76%
N 1.24%
EM 46.32%
EMRA 51.68%
CD107a
CD107a+ CD8+ T cells 60.77%
CD107a+ CD8dim T cells 84.46%
CD8dim CD107a+ T cells
CM 0.99%
N 2.72%
EM 9.23%
EMRA 87.07%
CD107a+ CD4+ T cells 2.09%
CD4+ CD107a+ T cells
CM 4.43%
N 1.01%
EM 86.53%
EMRA 8.03%

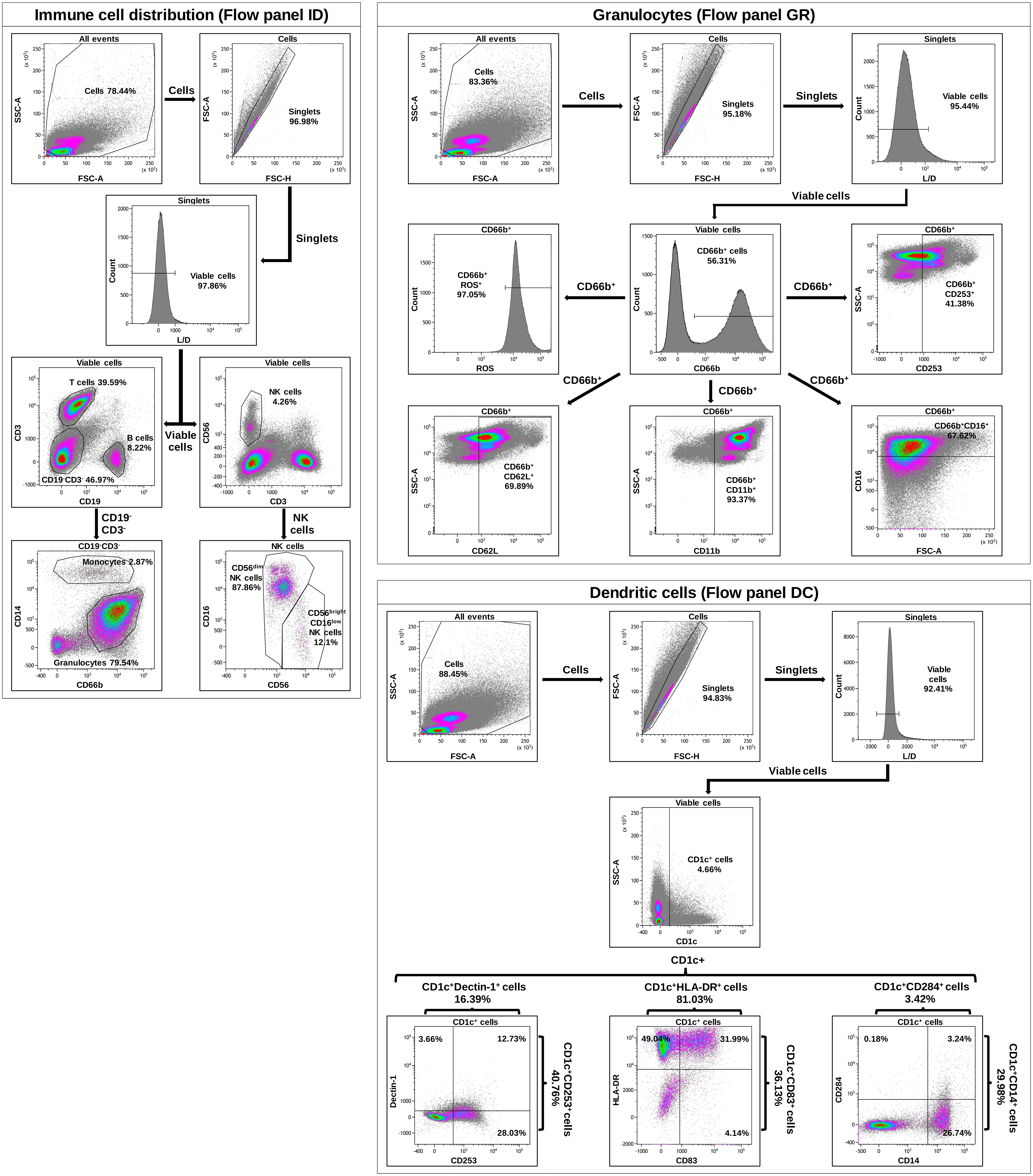

Immune cell distribution (Flow panel ID)
All events
Cells 78.44%
SSC-A
FSC-A
Cells
Singlets 96.98%
FSC-H
Singlets
Count
Viable cells 97.86%
L/D
Viable cells
T cells 39.59%
B cells 8.22%
CD19-CD3- 46.97%
CD3
CD19
NK cells 4.26%
CD56
Viable cells
CD19- CD3-
NK cells
CD19-CD3-
Monocytes 2.87%
Granulocytes 79.54%
CD14
CD66b
CD56dim NK cells 87.86%
CD56bright CD16low NK cells 12.1%
CD16
Granulocytes (Flow panel GR)
Cells 83.36%
Singlets 95.18%
Viable cells 95.44%
CD66b+ cells 56.31%
CD66b+
CD66b+ ROS+ 97.05%
ROS
CD66b+ CD253+ 41.38%
CD253
CD66b+ CD62L+ 69.89%
CD62L
CD66b+ CD11b+ 93.37%
CD11b
CD66b+CD16+ 67.62%
Dendritic cells (Flow panel DC)
Cells 88.45%
Singlets 94.83%
Viable cells 92.41%
CD1c+ cells 4.66%
CD1c
CD1c+
CD1c+Dectin-1+ cells 16.39%
CD1c+HLA-DR+ cells 81.03%
CD1c+CD284+ cells 3.42%
CD1c+ cells
3.66%
12.73%
28.03%
Dectin-1
CD1c+CD253+ cells 40.76%
49.04%
31.99%
4.14%
HLA-DR
CD83
CD1c+CD83+ cells 36.13%
0.18%
3.24%
26.74%
CD284
CD14
CD1c+CD14+ cells 29.98%
